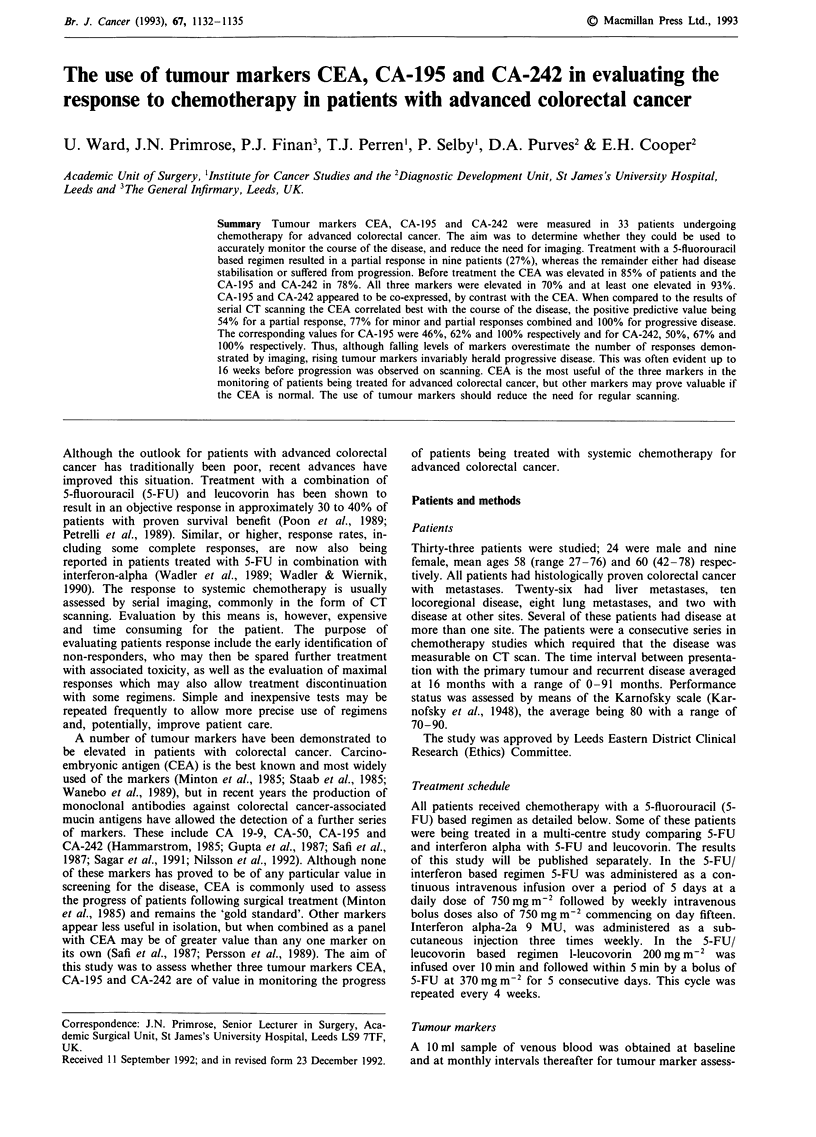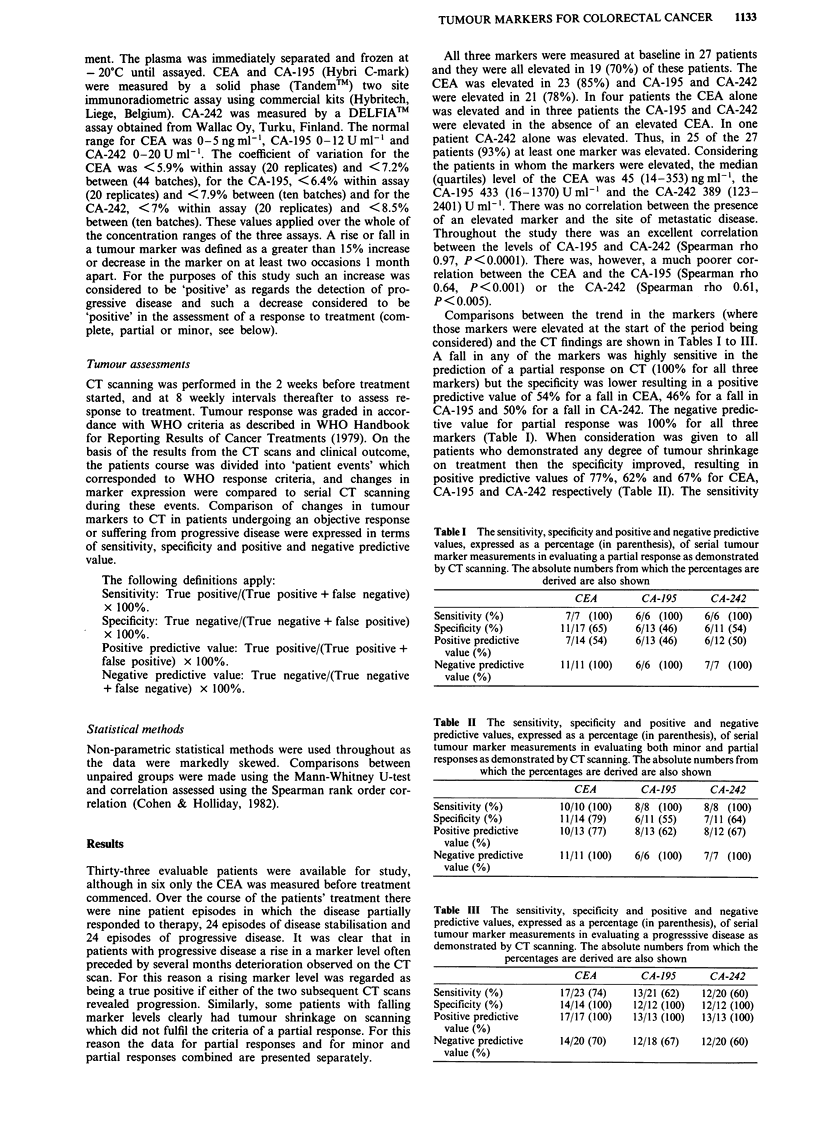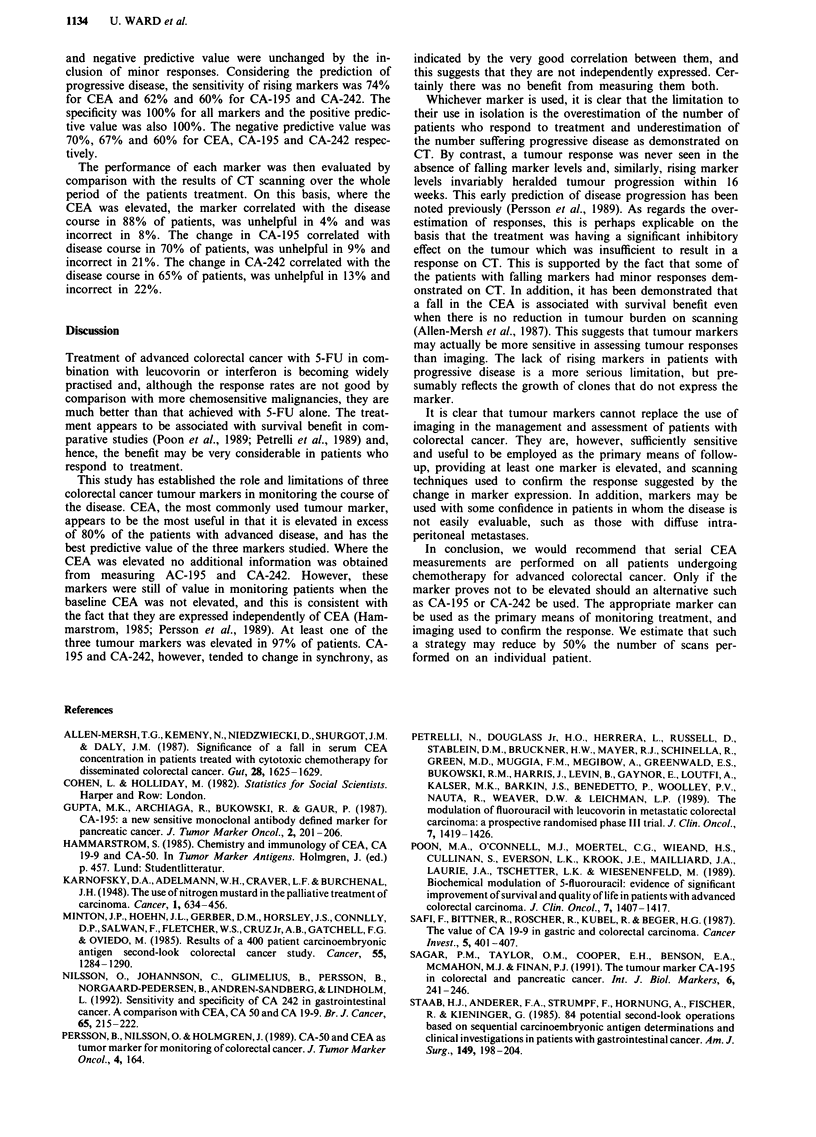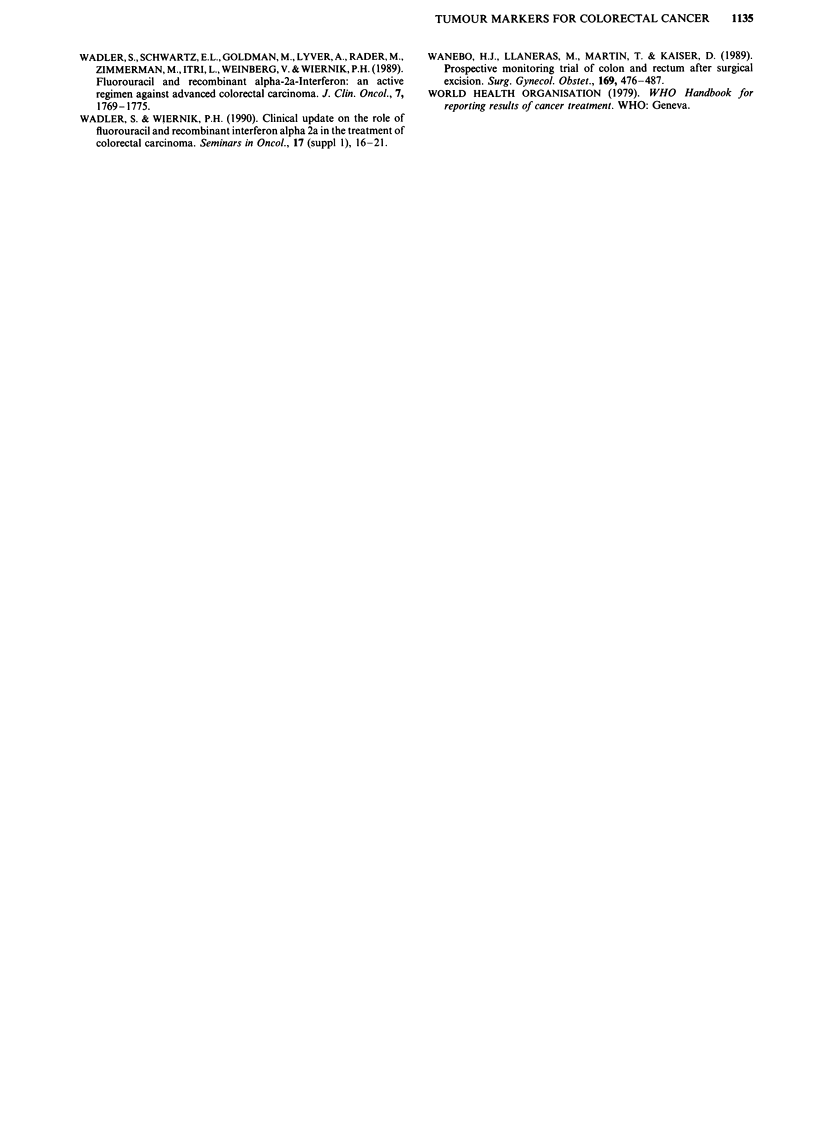# The use of tumour markers CEA, CA-195 and CA-242 in evaluating the response to chemotherapy in patients with advanced colorectal cancer.

**DOI:** 10.1038/bjc.1993.208

**Published:** 1993-05

**Authors:** U. Ward, J. N. Primrose, P. J. Finan, T. J. Perren, P. Selby, D. A. Purves, E. H. Cooper

**Affiliations:** Academic Unit of Surgery, St James's University Hospital, Leeds, UK.

## Abstract

Tumour markers CEA, CA-195 and CA-242 were measured in 33 patients undergoing chemotherapy for advanced colorectal cancer. The aim was to determine whether they could be used to accurately monitor the course of the disease, and reduce the need for imaging. Treatment with a 5-fluorouracil based regimen resulted in a partial response in nine patients (27%), whereas the remainder either had disease stabilisation or suffered from progression. Before treatment the CEA was elevated in 85% of patients and the CA-195 and CA-242 in 78%. All three markers were elevated in 70% and at least one elevated in 93%. CA-195 and CA-242 appeared to be co-expressed, by contrast with the CEA. When compared to the results of serial CT scanning the CEA correlated best with the course of the disease, the positive predictive value being 54% for a partial response, 77% for minor and partial responses combined and 100% for progressive disease. The corresponding values for CA-195 were 46%, 62% and 100% respectively and for CA-242, 50%, 67% and 100% respectively. Thus, although falling levels of markers overestimate the number of responses demonstrated by imaging, rising tumour markers invariably herald progressive disease. This was often evident up to 16 weeks before progression was observed on scanning. CEA is the most useful of the three markers in the monitoring of patients being treated for advanced colorectal cancer, but other markers may prove valuable if the CEA is normal. The use of tumour markers should reduce the need for regular scanning.


Br. J. Cancer (1993), 67, 1132–1135 © Macmillan Press Ltd., 1993

### The use of tumour markers CEA, CA-195 and CA-242 in evaluating the response to chemotherapy in patients with advanced colorectal cancer

U. Ward, J.N. Primrose, P.J. Finan[3], T.J. Perren[1], P. Selby[1], D.A. Purves[2] & E.H. Cooper[2]

*Academic Unit of Surgery, [1]Institute for Cancer Studies and the [2]Diagnostic Development Unit, St James's University Hospital, Leeds and [3]The General Infirmary, Leeds, UK.*

**Summary** Tumour markers CEA, CA-195 and CA-242 were measured in 33 patients undergoing chemotherapy for advanced colorectal cancer. The aim was to determine whether they could be used to accurately monitor the course of the disease, and reduce the need for imaging. Treatment with a 5-fluorouracil based regimen resulted in a partial response in nine patients (27%), whereas the remainder either had disease stabilisation or suffered from progression. Before treatment the CEA was elevated in 85% of patients and the CA-195 and CA-242 in 78%. All three markers were elevated in 70% and at least one elevated in 93%. CA-195 and CA-242 appeared to be co-expressed, by contrast with the CEA. When compared to the results of serial CT scanning the CEA correlated best with the course of the disease, the positive predictive value being 54% for a partial response, 77% for minor and partial responses combined and 100% for progressive disease. The corresponding values for CA-195 were 46%, 62% and 100% respectively and for CA-242, 50%, 67% and 100% respectively. Thus, although falling levels of markers overestimate the number of responses demonstrated by imaging, rising tumour markers invariably herald progressive disease. This was often evident up to 16 weeks before progression was observed on scanning. CEA is the most useful of the three markers in the monitoring of patients being treated for advanced colorectal cancer, but other markers may prove valuable if the CEA is normal. The use of tumour markers should reduce the need for regular scanning.

Although the outlook for patients with advanced colorectal cancer has traditionally been poor, recent advances have improved this situation. Treatment with a combination of 5-fluorouracil (5-FU) and leucovorin has been shown to result in an objective response in approximately 30 to 40% of patients with proven survival benefit (Poon *et al.*, 1989; Petrelli *et al.*, 1989). Similar, or higher, response rates, including some complete responses, are now also being reported in patients treated with 5-FU in combination with interferon-alpha (Wadler *et al.*, 1989; Wadler & Wiernik, 1990). The response to systemic chemotherapy is usually assessed by serial imaging, commonly in the form of CT scanning. Evaluation by this means is, however, expensive and time consuming for the patient. The purpose of evaluating patients response include the early identification of non-responders, who may then be spared further treatment with associated toxicity, as well as the evaluation of maximal responses which may also allow treatment discontinuation with some regimens. Simple and inexpensive tests may be repeated frequently to allow more precise use of regimens and, potentially, improve patient care.

A number of tumour markers have been demonstrated to be elevated in patients with colorectal cancer. Carcinoembryonic antigen (CEA) is the best known and most widely used of the markers (Minton *et al.*, 1985; Staab *et al.*, 1985; Wanebo *et al.*, 1989), but in recent years the production of monoclonal antibodies against colorectal cancer-associated mucin antigens have allowed the detection of a further series of markers. These include CA 19-9, CA-50, CA-195 and CA-242 (Hammarstrom, 1985; Gupta *et al.*, 1987; Safi *et al.*, 1987; Sagar *et al.*, 1991; Nilsson *et al.*, 1992). Although none of these markers has proved to be of any particular value in screening for the disease, CEA is commonly used to assess the progress of patients following surgical treatment (Minton *et al.*, 1985) and remains the 'gold standard'. Other markers appear less useful in isolation, but when combined as a panel with CEA may be of greater value than any one marker on its own (Safi *et al.*, 1987; Persson *et al.*, 1989). The aim of this study was to assess whether three tumour markers CEA, CA-195 and CA-242 are of value in monitoring the progress of patients being treated with systemic chemotherapy for advanced colorectal cancer.

Correspondence: J.N. Primrose, Senior Lecturer in Surgery, Academic Surgical Unit, St James's University Hospital, Leeds LS9 7TF, UK.
Received 11 September 1992; and in revised form 23 December 1992.

### Patients and methods

### *Patients*

Thirty-three patients were studied; 24 were male and nine female, mean ages 58 (range 27–76) and 60 (42–78) respectively. All patients had histologically proven colorectal cancer with metastases. Twenty-six had liver metastases, ten locoregional disease, eight lung metastases, and two with disease at other sites. Several of these patients had disease at more than one site. The patients were a consecutive series in chemotherapy studies which required that the disease was measurable on CT scan. The time interval between presentation with the primary tumour and recurrent disease averaged at 16 months with a range of 0–91 months. Performance status was assessed by means of the Karnofsky scale (Karnofsky *et al.*, 1948), the average being 80 with a range of 70–90.

The study was approved by Leeds Eastern District Clinical Research (Ethics) Committee.

### *Treatment schedule*

All patients received chemotherapy with a 5-fluorouracil (5-FU) based regimen as detailed below. Some of these patients were being treated in a multi-centre study comparing 5-FU and interferon alpha with 5-FU and leucovorin. The results of this study will be published separately. In the 5-FU/interferon based regimen 5-FU was administered as a continuous intravenous infusion over a period of 5 days at a daily dose of 750 mg $m^{-2}$ followed by weekly intravenous bolus doses also of 750 mg $m^{-2}$ commencing on day fifteen. Interferon alpha-2a 9 MU, was administered as a subcutaneous injection three times weekly. In the 5-FU/leucovorin based regimen l-leucovorin 200 mg $m^{-2}$ was infused over 10 min and followed within 5 min by a bolus of 5-FU at 370 mg $m^{-2}$ for 5 consecutive days. This cycle was repeated every 4 weeks.

### *Tumour markers*

A 10 ml sample of venous blood was obtained at baseline and at monthly intervals thereafter for tumour marker assessment. The plasma was immediately separated and frozen at − 20°C until assayed. CEA and CA-195 (Hybri C-mark) were measured by a solid phase (Tandem™) two site immunoradiometric assay using commercial kits (Hybritech, Liege, Belgium). CA-242 was measured by a DELFIA™ assay obtained from Wallac Oy, Turku, Finland. The normal range for CEA was 0–5 ng $ml^{-1}$, CA-195 0–12 U $ml^{-1}$ and CA-242 0–20 U $ml^{-1}$. The coefficient of variation for the CEA was <5.9% within assay (20 replicates) and <7.2% between (44 batches), for the CA-195, <6.4% within assay (20 replicates) and <7.9% between (ten batches) and for the CA-242, <7% within assay (20 replicates) and <8.5% between (ten batches). These values applied over the whole of the concentration ranges of the three assays. A rise or fall in a tumour marker was defined as a greater than 15% increase or decrease in the marker on at least two occasions 1 month apart. For the purposes of this study such an increase was considered to be 'positive' as regards the detection of progressive disease and such a decrease considered to be 'positive' in the assessment of a response to treatment (complete, partial or minor, see below).

*Tumour assessments*

CT scanning was performed in the 2 weeks before treatment started, and at 8 weekly intervals thereafter to assess response to treatment. Tumour response was graded in accordance with WHO criteria as described in WHO Handbook for Reporting Results of Cancer Treatments (1979). On the basis of the results from the CT scans and clinical outcome, the patients course was divided into 'patient events' which corresponded to WHO response criteria, and changes in marker expression were compared to serial CT scanning during these events. Comparison of changes in tumour markers to CT in patients undergoing an objective response or suffering from progressive disease were expressed in terms of sensitivity, specificity and positive and negative predictive value.

The following definitions apply:
Sensitivity: True positive/(True positive + false negative) × 100%.
Specificity: True negative/(True negative + false positive) × 100%.
Positive predictive value: True positive/(True positive + false positive) × 100%.
Negative predictive value: True negative/(True negative + false negative) × 100%.

*Statistical methods*

Non-parametric statistical methods were used throughout as the data were markedly skewed. Comparisons between unpaired groups were made using the Mann-Whitney U-test and correlation assessed using the Spearman rank order correlation (Cohen & Holliday, 1982).

### Results

Thirty-three evaluable patients were available for study, although in six only the CEA was measured before treatment commenced. Over the course of the patients' treatment there were nine patient episodes in which the disease partially responded to therapy, 24 episodes of disease stabilisation and 24 episodes of progressive disease. It was clear that in patients with progressive disease a rise in a marker level often preceded by several months deterioration observed on the CT scan. For this reason a rising marker level was regarded as being a true positive if either of the two subsequent CT scans revealed progression. Similarly, some patients with falling marker levels clearly had tumour shrinkage on scanning which did not fulfil the criteria of a partial response. For this reason the data for partial responses and for minor and partial responses combined are presented separately.

All three markers were measured at baseline in 27 patients and they were all elevated in 19 (70%) of these patients. The CEA was elevated in 23 (85%) and CA-195 and CA-242 were elevated in 21 (78%). In four patients the CEA alone was elevated and in three patients the CA-195 and CA-242 were elevated in the absence of an elevated CEA. In one patient CA-242 alone was elevated. Thus, in 25 of the 27 patients (93%) at least one marker was elevated. Considering the patients in whom the markers were elevated, the median (quartiles) level of the CEA was 45 (14–353) ng $ml^{-1}$, the CA-195 433 (16–1370) U $ml^{-1}$ and the CA-242 389 (123–2401) U $ml^{-1}$. There was no correlation between the presence of an elevated marker and the site of metastatic disease. Throughout the study there was an excellent correlation between the levels of CA-195 and CA-242 (Spearman rho 0.97, $P<0.0001$). There was, however, a much poorer correlation between the CEA and the CA-195 (Spearman rho 0.64, $P<0.001$) or the CA-242 (Spearman rho 0.61, $P<0.005$).

Comparisons between the trend in the markers (where those markers were elevated at the start of the period being considered) and the CT findings are shown in Tables I to III. A fall in any of the markers was highly sensitive in the prediction of a partial response on CT (100% for all three markers) but the specificity was lower resulting in a positive predictive value of 54% for a fall in CEA, 46% for a fall in CA-195 and 50% for a fall in CA-242. The negative predictive value for partial response was 100% for all three markers (Table I). When consideration was given to all patients who demonstrated any degree of tumour shrinkage on treatment then the specificity improved, resulting in positive predictive values of 77%, 62% and 67% for CEA, CA-195 and CA-242 respectively (Table II). The sensitivity

**Table I** The sensitivity, specificity and positive and negative predictive values, expressed as a percentage (in parenthesis), of serial tumour marker measurements in evaluating a partial response as demonstrated by CT scanning. The absolute numbers from which the percentages are derived are also shown

| | *CEA* | *CA-195* | *CA-242* |
|---|---|---|---|
| Sensitivity (%) | 7/7 (100) | 6/6 (100) | 6/6 (100) |
| Specificity (%) | 11/17 (65) | 6/13 (46) | 6/11 (54) |
| Positive predictive value (%) | 7/14 (54) | 6/13 (46) | 6/12 (50) |
| Negative predictive value (%) | 11/11 (100) | 6/6 (100) | 7/7 (100) |

**Table II** The sensitivity, specificity and positive and negative predictive values, expressed as a percentage (in parenthesis), of serial tumour marker measurements in evaluating both minor and partial responses as demonstrated by CT scanning. The absolute numbers from which the percentages are derived are also shown

| | *CEA* | *CA-195* | *CA-242* |
|---|---|---|---|
| Sensitivity (%) | 10/10 (100) | 8/8 (100) | 8/8 (100) |
| Specificity (%) | 11/14 (79) | 6/11 (55) | 7/11 (64) |
| Positive predictive value (%) | 10/13 (77) | 8/13 (62) | 8/12 (67) |
| Negative predictive value (%) | 11/11 (100) | 6/6 (100) | 7/7 (100) |

**Table III** The sensitivity, specificity and positive and negative predictive values, expressed as a percentage (in parenthesis), of serial tumour marker measurements in evaluating a progresssive disease as demonstrated by CT scanning. The absolute numbers from which the percentages are derived are also shown

| | *CEA* | *CA-195* | *CA-242* |
|---|---|---|---|
| Sensitivity (%) | 17/23 (74) | 13/21 (62) | 12/20 (60) |
| Specificity (%) | 14/14 (100) | 12/12 (100) | 12/12 (100) |
| Positive predictive value (%) | 17/17 (100) | 13/13 (100) | 13/13 (100) |
| Negative predictive value (%) | 14/20 (70) | 12/18 (67) | 12/20 (60) |

and negative predictive value were unchanged by the inclusion of minor responses. Considering the prediction of progressive disease, the sensitivity of rising markers was 74% for CEA and 62% and 60% for CA-195 and CA-242. The specificity was 100% for all markers and the positive predictive value was also 100%. The negative predictive value was 70%, 67% and 60% for CEA, CA-195 and CA-242 respectively.

The performance of each marker was then evaluated by comparison with the results of CT scanning over the whole period of the patients treatment. On this basis, where the CEA was elevated, the marker correlated with the disease course in 88% of patients, was unhelpful in 4% and was incorrect in 8%. The change in CA-195 correlated with disease course in 70% of patients, was unhelpful in 9% and incorrect in 21%. The change in CA-242 correlated with the disease course in 65% of patients, was unhelpful in 13% and incorrect in 22%.

### Discussion

Treatment of advanced colorectal cancer with 5-FU in combination with leucovorin or interferon is becoming widely practised and, although the response rates are not good by comparison with more chemosensitive malignancies, they are much better than that achieved with 5-FU alone. The treatment appears to be associated with survival benefit in comparative studies (Poon et al., 1989; Petrelli et al., 1989) and, hence, the benefit may be very considerable in patients who respond to treatment.

This study has established the role and limitations of three colorectal cancer tumour markers in monitoring the course of the disease. CEA, the most commonly used tumour marker, appears to be the most useful in that it is elevated in excess of 80% of the patients with advanced disease, and has the best predictive value of the three markers studied. Where the CEA was elevated no additional information was obtained from measuring AC-195 and CA-242. However, these markers were still of value in monitoring patients when the baseline CEA was not elevated, and this is consistent with the fact that they are expressed independently of CEA (Hammarstrom, 1985; Persson et al., 1989). At least one of the three tumour markers was elevated in 97% of patients. CA-195 and CA-242, however, tended to change in synchrony, as indicated by the very good correlation between them, and this suggests that they are not independently expressed. Certainly there was no benefit from measuring them both.

Whichever marker is used, it is clear that the limitation to their use in isolation is the overestimation of the number of patients who respond to treatment and underestimation of the number suffering progressive disease as demonstrated on CT. By contrast, a tumour response was never seen in the absence of falling marker levels and, similarly, rising marker levels invariably heralded tumour progression within 16 weeks. This early prediction of disease progression has been noted previously (Persson et al., 1989). As regards the overestimation of responses, this is perhaps explicable on the basis that the treatment was having a significant inhibitory effect on the tumour which was insufficient to result in a response on CT. This is supported by the fact that some of the patients with falling markers had minor responses demonstrated on CT. In addition, it has been demonstrated that a fall in the CEA is associated with survival benefit even when there is no reduction in tumour burden on scanning (Allen-Mersh et al., 1987). This suggests that tumour markers may actually be more sensitive in assessing tumour responses than imaging. The lack of rising markers in patients with progressive disease is a more serious limitation, but presumably reflects the growth of clones that do not express the marker.

It is clear that tumour markers cannot replace the use of imaging in the management and assessment of patients with colorectal cancer. They are, however, sufficiently sensitive and useful to be employed as the primary means of follow-up, providing at least one marker is elevated, and scanning techniques used to confirm the response suggested by the change in marker expression. In addition, markers may be used with some confidence in patients in whom the disease is not easily evaluable, such as those with diffuse intraperitoneal metastases.

In conclusion, we would recommend that serial CEA measurements are performed on all patients undergoing chemotherapy for advanced colorectal cancer. Only if the marker proves not to be elevated should an alternative such as CA-195 or CA-242 be used. The appropriate marker can be used as the primary means of monitoring treatment, and imaging used to confirm the response. We estimate that such a strategy may reduce by 50% the number of scans performed on an individual patient.

### References

ALLEN-MERSH, T.G., KEMENY, N., NIEDZWIECKI, D., SHURGOT, J.M. & DALY, J.M. (1987). Significance of a fall in serum CEA concentration in patients treated with cytotoxic chemotherapy for disseminated colorectal cancer. *Gut*, **28**, 1625-1629.

COHEN, L. & HOLLIDAY, M. (1982). *Statistics for Social Scientists*. Harper and Row: London.

GUPTA, M.K., ARCHIAGA, R., BUKOWSKI, R. & GAUR, P. (1987). CA-195: a new sensitive monoclonal antibody defined marker for pancreatic cancer. *J. Tumor Marker Oncol.*, **2**, 201-206.

HAMMARSTROM, S. (1985). Chemistry and immunology of CEA, CA 19-9 and CA-50. In *Tumor Marker Antigens*. Holmgren, J. (ed.) p. 457. Lund: Studentlitteratur.

KARNOFSKY, D.A., ADELMANN, W.H., CRAVER, L.F. & BURCHENAL, J.H. (1948). The use of nitrogen mustard in the palliative treatment of carcinoma. *Cancer*, **1**, 634-456.

MINTON, J.P., HOEHN, J.L., GERBER, D.M., HORSLEY, J.S., CONNLLY, D.P., SALWAN, F., FLETCHER, W.S., CRUZ Jr, A.B., GATCHELL, F.G. & OVIEDO, M. (1985). Results of a 400 patient carcinoembryonic antigen second-look colorectal cancer study. *Cancer*, **55**, 1284-1290.

NILSSON, O., JOHANNSON, C., GLIMELIUS, B., PERSSON, B., NORGAARD-PEDERSEN, B., ANDREN-SANDBERG, & LINDHOLM, L. (1992). Sensitivity and specificity of CA 242 in gastrointestinal cancer. A comparison with CEA, CA 50 and CA 19-9. *Br. J. Cancer*, **65**, 215-222.

PERSSON, B., NILSSON, O. & HOLMGREN, J. (1989). CA-50 and CEA as tumor marker for monitoring of colorectal cancer. *J. Tumor Marker Oncol.*, **4**, 164.

PETRELLI, N., DOUGLASS Jr, H.O., HERRERA, L., RUSSELL, D., STABLEIN, D.M., BRUCKNER, H.W., MAYER, R.J., SCHINELLA, R., GREEN, M.D., MUGGIA, F.M., MEGIBOW, A., GREENWALD, E.S., BUKOWSKI, R.M., HARRIS, J., LEVIN, B., GAYNOR, E., LOUTFI, A., KALSER, M.K., BARKIN, J.S., BENEDETTO, P., WOOLLEY, P.V., NAUTA, R., WEAVER, D.W. & LEICHMAN, L.P. (1989). The modulation of fluorouracil with leucovorin in metastatic colorectal carcinoma: a prospective randomised phase III trial. *J. Clin. Oncol.*, **7**, 1419-1426.

POON, M.A., O'CONNELL, M.J., MOERTEL, C.G., WIEAND, H.S., CULLINAN, S., EVERSON, L.K., KROOK, J.E., MAILLIARD, J.A., LAURIE, J.A., TSCHETTER, L.K. & WIESENENFELD, M. (1989). Biochemical modulation of 5-fluorouracil: evidence of significant improvement of survival and quality of life in patients with advanced colorectal carcinoma. *J. Clin. Oncol.*, **7**, 1407-1417.

SAFI, F., BITTNER, R., ROSCHER, R., KUBEL, R. & BEGER, H.G. (1987). The value of CA 19-9 in gastric and colorectal carcinoma. *Cancer Invest.*, **5**, 401-407.

SAGAR, P.M., TAYLOR, O.M., COOPER, E.H., BENSON, E.A., MCMAHON, M.J. & FINAN, P.J. (1991). The tumour marker CA-195 in colorectal and pancreatic cancer. *Int. J. Biol. Markers*, **6**, 241-246.

STAAB, H.J., ANDERER, F.A., STRUMPF, F., HORNUNG, A., FISCHER, R. & KIENINGER, G. (1985). 84 potential second-look operations based on sequential carcinoembryonic antigen determinations and clinical investigations in patients with gastrointestinal cancer. *Am. J. Surg.*, **149**, 198-204.

WADLER, S., SCHWARTZ, E.L., GOLDMAN, M., LYVER, A., RADER, M., ZIMMERMAN, M., ITRI, L., WEINBERG, V. & WIERNIK, P.H. (1989). Fluorouracil and recombinant alpha-2a-Interferon: an active regimen against advanced colorectal carcinoma. *J. Clin. Oncol.*, **7**, 1769–1775.

WADLER, S. & WIERNIK, P.H. (1990). Clinical update on the role of fluorouracil and recombinant interferon alpha 2a in the treatment of colorectal carcinoma. *Seminars in Oncol.*, **17** (suppl 1), 16–21.

WANEBO, H.J., LLANERAS, M., MARTIN, T. & KAISER, D. (1989). Prospective monitoring trial of colon and rectum after surgical excision. *Surg. Gynecol. Obstet.*, **169**, 476–487.

WORLD HEALTH ORGANISATION (1979). *WHO Handbook for reporting results of cancer treatment*. WHO: Geneva.